# Application of machine learning in dentistry: insights, prospects and challenges

**DOI:** 10.2340/aos.v84.43345

**Published:** 2025-03-27

**Authors:** Lin Wang, Yanyan Xu, Weiqian Wang, Yuanyuan Lu

**Affiliations:** a Hangzhou Stomatology Hospital, Hangzhou, China; b Health Service Center in Xiaoying Street Community, Hangzhou, China; c College of Environmental and Resources Sciences, Zhejiang University, Hangzhou, China

**Keywords:** Machine learning, dental practices, integration, diagnostics, treatment planning, patient management

## Abstract

**Background:**

Machine learning (ML) is transforming dentistry by setting new standards for precision and efficiency in clinical practice, while driving improvements in care delivery and quality.

**Objectives:**

This review: (1) states the necessity to develop ML in dentistry for the purpose of breaking the limitations of traditional dental technologies; (2) discusses the principles of ML-based models utilised in dental clinical practice and care; (3) outlines the application respects of ML in dentistry; and (4) highlights the prospects and challenges to be addressed.

**Data and sources:**

In this narrative review, a comprehensive search was conducted in PubMed/MEDLINE, Web of Science, ScienceDirect, and Institute of Electrical and Electronics Engineers (IEEE) Xplore databases.

**Conclusions:**

Machine Learning has demonstrated significant potential in dentistry with its intelligently assistive function, promoting diagnostic efficiency, personalised treatment plans and related streamline workflows. However, challenges related to data privacy, security, interpretability, and ethical considerations were highly urgent to be addressed in the next review, with the objective of creating a backdrop for future research in this rapidly expanding arena.

**Clinical significance:**

Development of ML brought transformative impact in the fields of dentistry, from diagnostic, personalised treatment plan to dental care workflows. Particularly, integrating ML-based models with diagnostic tools will significantly enhance the diagnostic efficiency and precision in dental surgeries and treatments.

## Introduction

**Figure 1:**
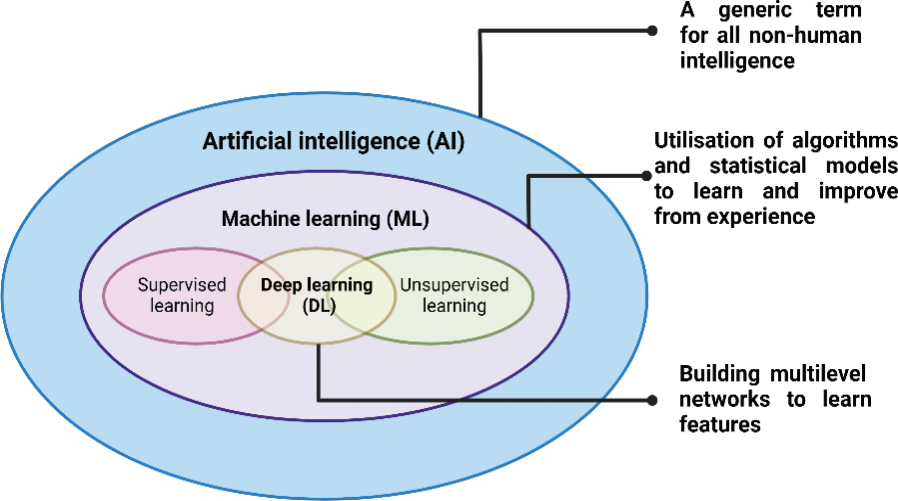
Relationship and concepts among Artificial Intelligence, Machine Learning, and Deep Learning.

The terms such as ‘artificial intelligence’ (AI), ‘machine learning’ (ML) and ‘deep learning’ (DL) appeared more and more frequently in various industries including finance [1], manufacture [2], retail [3], and healthcare [4] in the late 5 years. In the field of healthcare, dentistry is also undergoing revolutionised transitions towards their adoption, from diagnosis, treatment to administrative management [5], facilitating the development of rapid and efficient characterisation [6, 7] and prediction [8] in a variety of health risks. The simplest way to think of their relationship is to consider them as concentric circles (shown in Figure 1). After understanding clearly their differences and contributions in health care, this review seeks to illuminate the potential of ML in a brighter future for dentistry. Relative to traditional programming, the application of ML helps to solve complex tasks without specific reprogramming in each scenario, enhancing the performance in adapting to new-input data and identifying patterns [9].

Machine Learning mainly encompasses four popular approaches including supervised learning [10, 11], unsupervised learning [12], semi-supervised learning [13]. and reinforcement learning [14] designed for addressing various specific problems. Deep Learning acts as a unifying technique, enhancing performance across all these paradigms by leveraging the power of deep neural networks. Notably, application potential of ML in dentistry has attracted wide attention, considering that traditional dental diagnostics and treatment planning relied tightly on the clinician’s expertise and manual image analysis. The revolutionised integration of ML into dentistry is conducted by employing advanced models such as artificial neural networks (ANNs) [15], convolutional neural networks (CNNs) [16], and support vector machines (SVMs) [17]. These models can help in analysing diverse types of data from dental practices, including high-resolution images like dental radiographs, intraoral and three-dimensional (3D) scans, and historical patient records [18, 19]. Accordingly, the patient experience will be optimised through the personalisation of various aspects, including appointment scheduling and environmental preferences during visits. In addition, patient management, driven by predictive analytics based on ML, will lead to more comfortable and tailored experiences [20]. By proactively addressing these factors, dental practices can enhance adherence to treatment plans, improve patient satisfaction, and increase overall cost-effectiveness.

Notably, the integration of ML in dentistry is still facing huge challenges. Before ensuring the safety and effective use of ML in dental care to great degree, issues related to data privacy, security, ethical considerations, and need for human oversight — those essential for the successful adoption and further advancement of ML in dentistry — are necessary to be solved. Herein, this review aims to explore the development of ML application in dentistry by illustrating the diverse functions in dental practice, highlighting the benefits of ML application offered in diagnostic, treatment planning, and patient management, providing prospects for the future development of ML including potential innovations and on-going evolution of dental practices. Furthermore, challenges associated with the integration of ML into current-applied dental technologies and conditions are addressed. Through the above-mentioned exploration, the review seeks to offer a comprehensive understanding of how ML application transforms the dentistry field at present and in the future, which not only contributes to set new standards for precision and efficiency in clinical practice but also marks a paradigm shift in the delivery and quality improvement in dental care.

## Necessity to develop Machine Learning in dentistry

The advancement of technologies updated in dentistry has been a progressive journey, characterised by a series of innovations that have fundamentally transformed the dental practice [21]. These advancements initially focussed on enhancing diagnostic accuracy, improving the precision of treatments, and streamlining the operations of dental practices. With the technical evolution, more sophisticated applications emerged but also underlined the importance of integrating ML in modern dental care. Therefore, this section first provides a comprehensive overview of the early technological milestones in dentistry, describes the corresponding limitations from traditional methods that ML-based approaches can help to break, clarifying the necessity of developing ML due to its profound impact in this field.

### Dental radiography

The advent of dental radiography in the early 20th century marked a milestone moment in dental diagnostics, especially the introduction of X-ray technology allowed dentists to visualise the internal structures of teeth and gums, enabling the detection conditions previously invisible to the naked eyes. The pioneering work enhanced diagnostic accuracy with reduced radiation exposure, and real-time and high-resolution image acquisition provided critical insights into cavities, bone loss, and other pathologies [22, 23]. A more advanced assessment technique—Cone Beam Computed Tomography (CBCT) in the late 1990s and early 2000s provided 3D imaging capabilities that were particularly useful for complex diagnostic and surgical planning, which is essential in implantology and orthodontics to offer detailed 3D views of the teeth, bone structures, and soft tissues [24, 25].

However, the existing limitations in dental radiography are obvious, the biggest problem is the inconsistent diagnoses, a lack of standardisation and potential misinterpretations of radiographic images from the evaluation variability interpreted by human experts such as radiologists or dentists based on their experience and training, sometimes depending on their physical and mental state at the time of assessment. Minor and subtle changes from certain dental conditions such as early-stage caries, microcracks, or initial signs of periodontal disease are challenging for even experienced practitioners to detect, leading to the delay in diagnosis and treatment. In fast-paced clinical environments currently, it is highly urgent for immediate diagnostic support to make quick and accurate decisions in dental emergencies or during complex procedures.

### Dental drills and lasers

The development of high-speed dental drills in mid-20yh century was another transformative progress in modern dentistry, improving the efficiency of dental procedures such as cavity preparation and removal of decayed tissue [26]. In 1980s, the introduction of dental lasers further advanced dental procedures and marked an improvement in patient care which was initially used for soft tissue operations such as gum reshaping then gradually further applied in hard tissue procedures, offering a less invasive and painful alternative to traditional drills and scalpels [27, 28]. However, limited precision in depth and location control of dental drills still need to be discussed; although dental lasers offer more precision than traditional drills, compounded by human error, fatigue or limited visibility in difficult-to-reach areas, leading to the removal of non-target tooth tissues than necessary. Mechanical vibration, heat generation, and accumulation will cause discomfort and even unintended damage such as burns or tissue necrosis without proper management [29]. Meanwhile, the limitation from incomplete integration into digital workflow made them unable to benefit from the latest digital techniques such as guided surgery, computer-aided design and manufacturing (CAD/CAM).

### Computer-aided design and manufacturing

The introduction of CAD/CAM technology in dentistry is primarily for designing and fabricating dental restorations such as crowns, bridges, and veneers [30]. This technology enabled the production of more precise and aesthetically pleasing restorations within a single visit. Through incorporating with 3D imaging and advanced materials, CAD/CAM further enhanced their adoption in restorative and prosthodontic procedures from dental laboratories and clinics, validating their importance in modern dental practice [31, 32]. Computer-aided design software often struggles with complex multiparameter optimisation because of the sheer number of variables and constraints, requiring extensive manual intervention to find the best solution. As for CAM, recognising and categorising features (like holes, slots or pockets) from CAD models is crucial for machining. However, traditional CAM software may struggle with complex or unconventional geometries, bringing errors in manufacturing. Besides, tool paths generated by deterministic algorithms from CAM software may not consider the time and energy consumption, as well as the optimal material properties and process parameters (like temperature, feed rate, and speed), plus with the repeated trial and error caused by human.

### Computer-based streamlining operations

The end of 20th century witnessed the development of computer-based practice management software, which significantly streamlined dental practice operations [33]. These early systems managed scheduling, billing, and patient records, reducing the administrative burden towards dental staff and facilitated the communication with patients. The evolution of these systems into comprehensive electronic health records (EHRs) allowed for the integration of diagnostic data, treatment plans, and medical records of patients, thereby promoting better coordination of dental care and overall practice efficiency [34].

But the key limitation lies in the difficulty in processing and interpreting large volumes of unstructured data from various sources like clinical notes, X-rays and patient historic medical records, leading to inefficiencies and incomplete insights. Another is the lack of predictive capabilities in current systems, which hampers the ability to foresee patient outcomes, optimise scheduling, or anticipate the needs for equipment maintenance. In addition, traditional software struggles with adapting to the nuances of individual patient care, only relying on rigid protocols that may not account for unique patient variables.

### Orthodontic technologies

Orthodontics also benefited from technological advancements, particularly with the introduction of cephalometric analysis [35, 36] and clear aligners [37, 38]. Cephalometric analysis, which utilised radiographs to measure the relationships between the teeth, jaw, and skull, allowed orthodontists to develop more accurate treatment plans. The late 1990s witnessed the emergence of clear aligners, offering a more aesthetic and comfortable alternative to traditional braces. These innovations in orthodontics not only improved treatment outcomes but also enhanced patient satisfaction. But similar with other dental technologies, the major limitation for orthodontic technologies is also inconsistent outcomes, prolonged treatment times, and suboptimal results due to the reliance on manual diagnosis and treatment planning with significant variability. While the difficulty in predicting patient adherence and biological response to treatments like aligners or braces is another problem to be solved for traditional methods. Current orthodontic tools often struggle with real-time monitoring in treatment progress, they have no way to provide dynamic adjustments based on ongoing changes of patient’s condition; although some systems are equipped with rudimentary tracking.

### The transition from traditional methods to ML-based approaches

**Figure 2:**
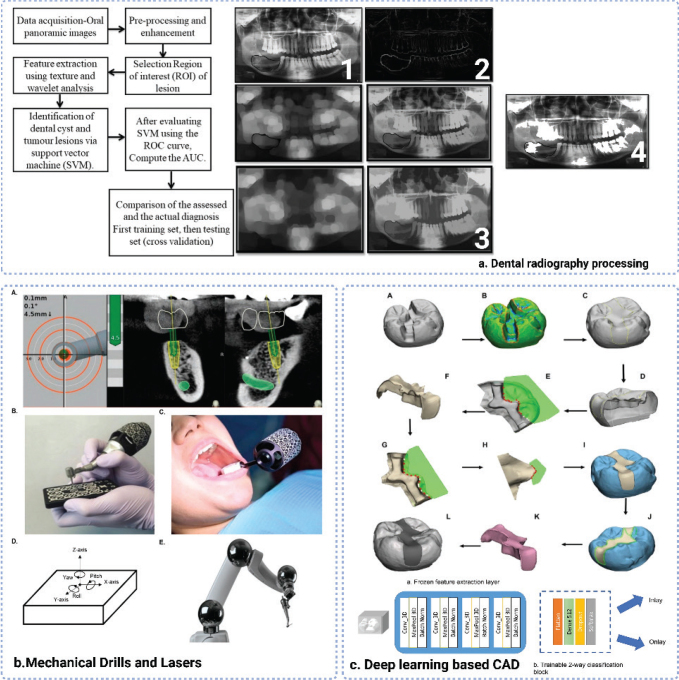
Based on varies Machine Learning approaches: (a) dental panoramic radiographs processing schemes and corresponding representation of morphological processing [47]; (b) mechanical drills and lasers for computer-navigated implant dynamic surgery [48]; (c) 3-dimensional convolutional neural network (3D-CNN) to generate partial dental crowns (PDC) for use in restorative dentistry [49].

Since the appearance of Computer-Aided Detection systems in the early 2000s [39], ML was initially applied in dental radiology as a key start point in the development. Employing basic algorithms to assist dentists in detecting dental abnormalities in digital radiographs, setting the stage for more and more sophisticated ML applications. The widespread utilisation of digital tools allowed the availability of large datasets to speed up the development of various ML algorithms in the late 2000s [40]. During this period, research focussed on improving the accuracy of caries and periodontal disease diagnosis, paving the way for the clinical applications of ML in dentistry [41]. Then in 10 years, it was seen that more advanced ML algorithms such as SVM [42] and ANN [43] started to play a function in dental imaging. In particular, the exploit of DL further enhanced the capability of ML models to perform complex tasks in image analysis, leading to more accurate diagnostics [44, 45]. By the mid-2010s, the feasibility of CNNs was confirmed in dental diagnostics, further offering higher accuracy in tasks such as cavity detection, periodontal disease diagnosis, and oral cancer screening [46]. After that, the commercialisation and widespread adoption of ML-based dental tools made a significant shift in dental care, combining with diagnostics, treatment planning, and practice management (Figure 2).

To solve the limitations correspondingly mentioned in the last section, the transition from traditional methods to ML-based approaches in dentistry absolutely is a big innovation, moving from manual, experience-based practices to data-driven and automated systems. In contrast to traditional methods, ML-based approaches held the increased accuracy, consistency, and predictive capabilities enabling more precise diagnostics, personalised treatment plans, and streamlined practice operations; breaking the limits from the variability of human interpretation and the lack of personalised treatment options. Particularly, the emergence of large-scale AI models [50], including large language models (LLMs), large vision models (LVMs), and large multimodal models (LMMs) has further expanded the application scope of AI in dentistry. The integration of these advanced models with existing ML-based systems is expected to enhance diagnostic precision, streamline clinical workflows, and foster deeper patient engagement. For this part, we will discuss more details in the section 3.

## Current applications of machine learning in dentistry

Limitations in technologies towards various aspects of dentistry as indicated in ‘Necessity to develop ML in dentistry’ section hampered the advancement in the fields of dental diagnostics, treatment planning, predictive analytics, and even integration with dental robotics, but a new era in dentistry driven by AI brings ML approaches to offer innovative solutions for those long-standing challenges. Next, we will give a detailed exploration of the key applications of ML in dentistry at present towards corresponding problems faced by traditional dental methods, highlighting its deep impact for dental diagnostic and treatments.

### Principles of Machine Learning application in dentistry

**Figure 3:**
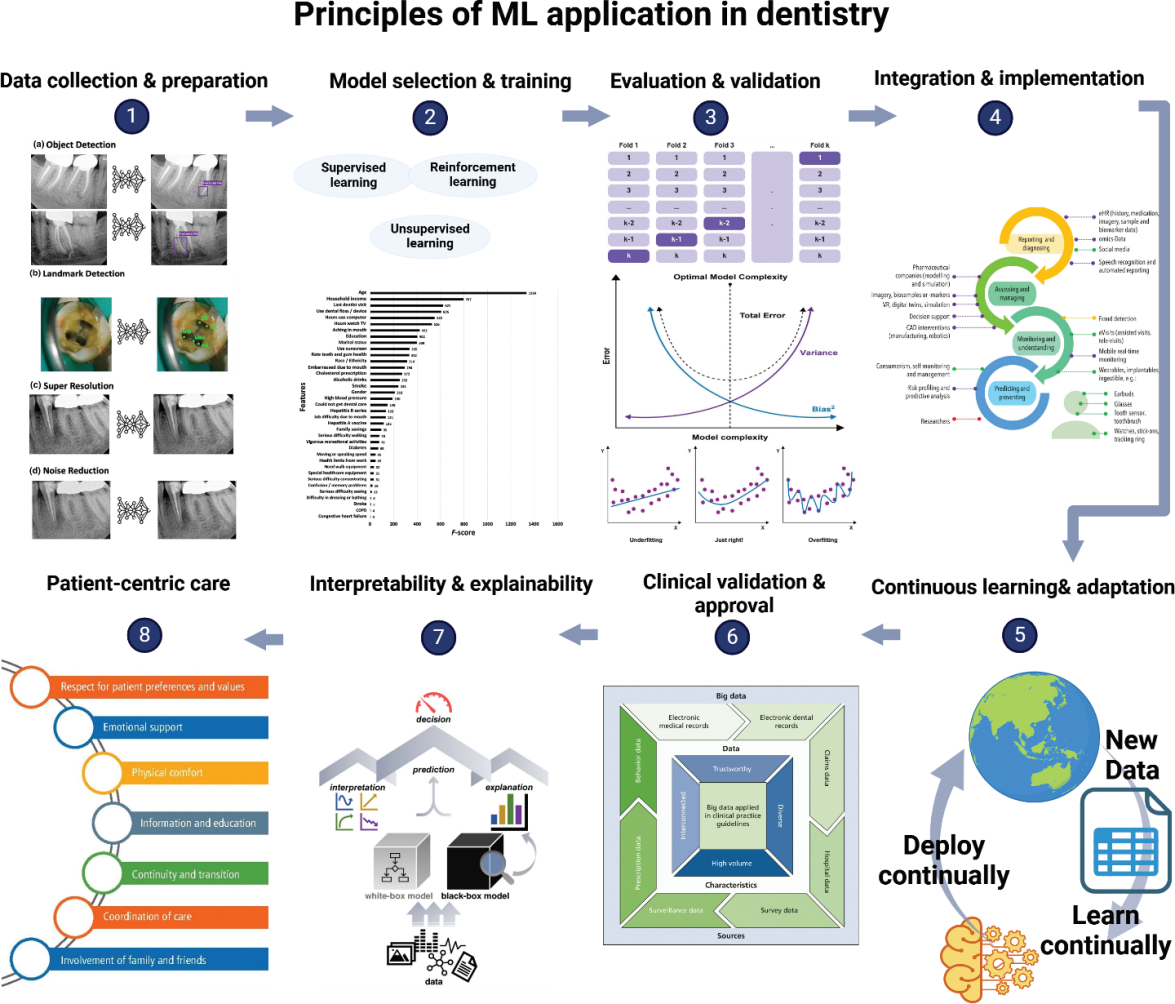
Schemic illustration for principles of Machine Learning application in dentistry, images utilised inside from literatures [18, 52–56].

Embedding ML into diagnostic tools, procedures, and workflows involves training data-driven algorithms on extensive dental datasets. How to recognise patterns, anomalies, and correlations within the data normally follows principles to face the significant heterogeneity in the data, tasks, models, and performance metric [51]. Herein, procedures that combine statistical and computational patterns in the analysis and critical interpretation of datasets are introduced in this section (as depicted in Figure 3), to obtain valid criteria for ML applications in dentistry.

### Early diagnosis

**Figure 4:**
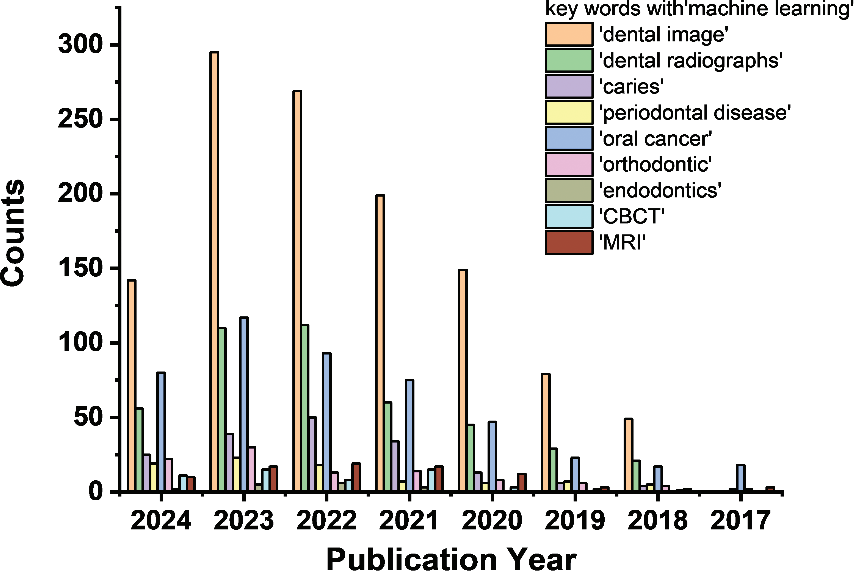
Publication trends from 2017 to 2024 with different key words related to Machine Learning-based dental diagnosis as counted from Web of Science.

Focussing on early detection, the ML-based approaches play the critical roles in the detection of dental caries [57–59], periodontal diseases [53, 60], oral cancer [61], orthodontic [62], endodontics [63], dental anomalies [64] (impacted or supernumerary teeth), and so on (yearly publication amount as presented in Figure 4). In comparison with traditional methods, ML models offer higher accuracy and consistency as they can automatically process and analyse a large quantity of dental radiographs, through learning the patterns and characteristics associated with certain dental condition, identifying signs that might not be visible for dentists’ eyes with reliable diagnostic results. Different ML models are applied in various dental symptoms, based on various types of algorithms to analyse radiographs (panoramic X-ray [65], periapical [66], cephalometric [67], etc.), intraoral [68] or histopathological images [69], facilitating timely intervention, and development of personalised preventive strategies. Moreover, predictive and differentiate analytics on the management of temporomandibular disorders (TMD) [70] or progression [71] of dental diseases such as periodontal and apical lesions (cysts, granulomas and abscesses) also guide clinicians in selecting the most appropriate treatment plans tailored to individual patients. Recent advancements in LVMs are redefining the capabilities of AI-driven dental diagnostics. Unlike traditional CNN-based models, LVMs such as Segment Anything Model (SAM) [72] and Vision Transformers (ViTs) [73] can autonomously segment teeth, identify dental anomalies, and classify oral diseases with minimal reliance on labelled training data. These models after extensive pre-training on diverse imaging datasets, significantly mitigate the dependency on human-annotated dental images, as a major limitation in conventional ML models.

### Integration with Advanced Diagnostic Tools

Integration of ML with advanced diagnostic tools like CBCT [74] and Magnetic Resonance Imaging (MRI) [75] will further augment its practicability in dental diagnostics. Not at least for the early diagnostic, advancement of ML models can also help to detect subtle patterns and anomalies from dental radiographs or even with the help of real-time monitoring tools, providing immediate insights during the significant procedures such as implant placements or endodontic surgeries. LMMs represent a breakthrough in personalised dentistry by integrating diverse patient data sources [76]. Unlike traditional ML models that rely on single-modality data (e.g. X-rays or textual records), LMMs can simultaneously process text (patient history), images (X-rays, CBCT scans), and even genetic information; thereby enabling data-driven, accurate and standardised dental diagnostics.

### Personalised treatment planning

Machine Learning also significantly contributes to personalised treatment planning across various dental disciplines. In orthodontics, ML could analyse the historical data to optimise the treatment plans, predict tooth movements, and estimate treatment duration; thereby improving the overall treatment efficiency [77]. In prosthodontics, ML aids in designing custom dental prosthetics based on a patient’s unique oral health and anatomy. The success of tooth alignment in orthodontics or the durability of prosthetics in prosthodontics validated the feasibility of predicting treatment outcomes from ML-driven analytics, allowing clinicians to make pre-emptive adjustments to enhance patient care. Multimodal transformers such as GPT-4V and Meta’s LLaMA can analyse dental charts, past treatment outcomes, and imaging data to generate personalised recommendations for orthodontic aligners, prosthetic restorations, and implant placements [78].

### Predictive analytics for dental restoration and maintenance

In the field of dental restoration and maintenance [79], ML applications have become increasingly important in predictive analytics. According to individual patient’s data, the longevity of dental restorations can be estimated using suitable predictive models to assist the selection of appropriate dental materials and treatment planning. The models continuously keep pace with the updated-input data, enhancing their predictive accuracy and relevance in clinical settings. Meanwhile, ML-based monitoring tools are employed to track orthodontic treatment process so as to make necessary adjustments in time for improved outcomes. As for the prosthodontics, ML-based approach helps to make decision on when and whether the maintenance or replacement is required for dental prosthetics, meeting patients’ satisfaction from the enhanced treatment level.

### Integration of Machine Learning in robotic dental surgery and automated procedures

In the field of robotic dental surgery and automation of various dental procedures, ML algorithms helped to guide robotic systems in performing precise procedures, such as implant placements and tooth extractions, reducing complications, and improving patient outcomes [80]. In endodontics [81], ML-powered robotics automate procedures like root canals; while in orthodontics, they streamline tasks such as bracket placement, enhancing accuracy, and efficiency. In prosthodontics [80], ML improves robotic milling machines used for fabricating dental prosthetics by analysing data on successful restorations, ensuring better fit and durability. Large Language Models and LVMs can enable real-time surgical guidance, anomaly detection, and adaptive decision-making in robotic-assisted procedures. For instance, LLMs such as GPT [82] or MedPaLM [83] can serve as intelligent surgical assistants, providing voice-controlled guidance, intraoperative documentation, and patient-specific real-time recommendations to support clinicians during complex procedures. Meanwhile, LVMs can enhance robotic vision by enabling real-time segmentation of anatomical structures, ensuring greater accuracy in procedures such as implant placements, root canal treatments, and maxillofacial surgeries. In addition, reinforcement learning-driven robotic systems can continuously improve their precision over time by learning from past surgical outcomes, reducing procedural variability and improving long-term patient outcomes.

## Challenges and prospects

Despite ML offering numerous benefits in dentistry, the integration of ML into clinical practice inevitably faces challenges and limitations to be carefully considered. Tackling these factors will help to make dental ML technologies better and facilitate their uptake in clinical care.

### Data availability and quality

One of the primary challenges in implementing ML in dentistry is the quality and availability of data considering the requirements for large, high-quality, and comprehensive datasets to function more effectively in existing clinical systems. However, incomplete, inconsistent, and biased data because of variations in documentation practices across varied clines also brings a lot of problem. Most of all, limited availability of well-annotated datasets, particularly for diagnostic purposes, hampering the development of robust and reliable ML models to great degree in dentistry, especially for vast amounts of sensitive patient data to be managed [84]. Accordingly, ensuring data privacy, security, and compliance with regulations is essential in real-time processing and cloud-based storage.

### Interpretability and transparency

As mentioned in Section 3 above, the interpretability of complex ML models remains a challenge, requiring explainable AI techniques to provide clinicians with transparent and understandable predictions. Thus, it is essential for dental professionals to take necessary and adequate training on the adoption of ML-based tools. On the other hand, advanced ML models, especially DL models, are often considered as ‘black boxes’ due to their decision-making processes not easily interpretable. The lack of transparency can make it difficult to persuade clinicians in trusting the accuracy of predictions from models although built on evidence-based practice [85], particularly for those critical treatment decisions. Ensuring ML models to be explainable and transparent is a significant challenge; for effectively integrating the model-based practice into patient care and maintaining the professional accountability, clinicians must better understand the recommendation derived from underlying evidence, reasoning, and context.

### Integration with clinical workflow

Integrating ML into existing dental practices is still disruptive and challenging currently considering the lack of workflows to be established and additional training for dental professionals. Moreover, achieving seamless interoperability between ML systems and existing dental software or EHRs [53] is technically complex and resource-intensive, potentially slowing down the adoption of these technologies.

### Ethical and legal considerations

The use of ML in dentistry has raised ethical and legal concerns in the society, particularly regarding data privacy and security. With the increasing reliance on cloud-based solutions, more robust safeguards are highly urgent for preventing the breaches of sensitive patient data in the management. While given that the blurred line of responsibility among clinicians, developers and healthcare organisations, the accountability to be determined in cases where the harm lead by ML-driven decisions on patients’ health remains a key legal challenge.

As the continued advancements in ML algorithms (DL, reinforcement learning, and ensemble methods), the focus of developing ML in dentistry still lies in improving the efficacy and accuracy of predictive analytics on oral health outcomes. By considering more diverse data source such as clinical records, genetic information and social determinant such as lifestyle factors, a comprehensive understanding for oral health problems will be provided based on the enhanced predictive power of model. In addition, ML will increasingly facilitate the personalised and precision dentistry, where treatment plans are tailored not only to patient-specific data but also adapted as real-time as data collection. This integrated system will leverage genomics to predict the influence from patient’s genetic makeup on treatment outcomes, offering highly individualised care that responds dynamically to the patient’s ongoing health status. Meanwhile, development of real-time monitoring systems and decision support tools can enable the early intervention and timely adjustment of treatment plans to optimise the patient outcomes beyond traditional dental care.

On the other hand, the integration of ML with augmented reality (AR), virtual reality (VR), and wearable technology [86] will also enhance both diagnostic and procedural accuracy in dentistry. Machine Learning-driven AR could assist dentists throughout the surgery process by providing real-time overlays of crucial anatomical information, while wearable smart devices could enable continuous monitoring of oral health, feeding real-time data back to ML systems for ongoing analysis and intervention. Besides, the role of ML in developing autonomous and semi-autonomous robotic systems [87] in dentistry is expected to transform the dental procedures. The integrated systems could perform routine tasks with minimal human intervention, ensuring consistency, reduced errors, and more accessible dental care. Semi-autonomous robots [88] could work alongside dentists, providing dynamic support during complex procedures, thus enhancing precision and patient outcomes. Meanwhile, future research should prioritise the development of dental-specific large AI models, enhancing resource efficiency and integrating explainable AI (XAI) techniques to improve model transparency. In addition, federated learning offers a promising approach for privacy-preserving AI training, enabling secure collaboration across dental institutions while safeguarding sensitive patient data.

## Conclusions

Overall, applying ML-based approaches into diverse fields of dentistry leads to more accurate diagnoses, personalised treatment plans, and more efficient practice management. As ML technology continues to evolve, its potential applications in dentistry are expected to expand and offer greater benefits in terms of patient care, preventive strategies, and operational efficiency. This technological advancement not only enhances the dental professionals but also improves the overall patient experience from more precise, personalised, and proactive dental treatment.

## Conflicts of interest

The authors declare that they have no known competing financial interests or personal relationships that could have appeared to influence the work reported in this paper.

## Supplementary Material

Application of machine learning in dentistry: insights, prospects and challenges
