# Supplementary material for: Application of machine learning in dentistry: insights, prospects and challenges
Source: Acta Odontol Scand. 2025 Mar 27;84:43345. doi: 10.2340/aos.v84.43345 (PMC11971948; doi:10.2340/aos.v84.43345)
Supplement: Application of machine learning in dentistry: insights, prospects and challenges [file AOS-84-43345-s1.pdf]

Supplementary material has been published as submitted. It has not been copyedited or typeset by Acta Odontologica Scandinavica.

Application of Machine Learning in Dentistry: Insights and Challenges

-Supplementary Material-

Lin Wang [a], Yanyan Xu [b], Weiqian Wang [a, *], Yuanyuan Lu [c, *]

[a] *Hangzhou Stomatology Hospital, Hangzhou 310063, China*

[b] *Health Service Center in Xiaoying Street Community, Hangzhou 310009, China*

[c] *College of Environmental and Resources Sciences, Zhejiang University, Hangzhou 310058, China*

* Corresponding author:

Weiqian Wang (Email: hzwwq@163.com)

Yuanyuan Lu (Email: yuanyuan.lu@zju.edu.cn)

Section 1 Principles of ML application in dentistry

a. Data collection and preparation

-Data Quality and Quantity: data collected from various sources including digital radiographs, cone-beam computed tomography (CBCT) scans, intraoral photographs, 3D models, patient medical records and clinical notes should be findable, accessible, interoperable and reusable to be used in ML models [1].

-Data Preprocessing: raw dental data often needs to be cleaned, normalized and preprocessed to eliminate the noise and fill missing values, ensuring the uniformity. This step introduces data augmentation techniques to diversify the dataset and improve model generalization [2, 3].

-Data Labeling: The accuracy of the labels is essential for ML subsequent reliable use in the clinical setting, this involves annotating images (e.g., identifying areas of decay or lesions), categorizing diagnoses or labeling treatment outcomes based on experts' opinions or clinical records [4].

b. Model selection and training

-Algorithm selection: choosing the right ML algorithms that match the specific application in dentistry. Common algorithms are listed as follows:

1)Supervised learning: including decision trees [5], SVMs [6] and neural networks [7] mainly used for diagnosis, prognosis and classification of dental conditions.

2)Unsupervised learning: including k-means clustering [8] and hierarchical clustering [9] techniques used for data segmentation such as grouping patients based on similar risk profiles or treatment needs.

3)Reinforcement learning: used for developing optimal treatment plans [10] or robotic-assisted surgeries [11], where models are continuously adapted and improved by receiving feedback from patient responses or treatment effectiveness.

-Model training: feeding the ML model with labeled data to learn patterns, relationships and features associated with specific dental conditions or treatment outcomes.

-Feature engineering: selecting or deriving the most relevant features from the dataset to achieve accurate model predictions. This might involve identifying specific attributes from dental images or extracting significant clinical information from patient medical records [12].

c. Model evaluation and validation

-Performance metrics: to assess the accuracy and reliability of ML models, common metrics including accuracy, precision, sensitivity, F1-score and ROC-AUC (Receiver Operating Characteristic - Area Under the Curve) will help to evaluate the reliability and clinical utility of algorithms for identifying, diagnosing or predicting under certain dental conditions [13].

-Cross-validation: techniques like K-fold cross-validation [14-16] are used to validate the model's performance across different subsets of the data.

- Bias and variation: regularization and hyperparameter tuning in managing the bias-variance tradeoff is to prevent model underfitting or overfitting help, so as to optimize model performance [17].

d. Integration and implementation

-Workflow integration: integrating ML models into existing dental workflows could provide the complement of clinical practices without disruption (e.g. diagnostic imaging software for real-time decision support) [18].

-User interface design: the output of ML models should be presented in a user-friendly manner so that dental specialists can interpret and act on the provided information more easily, such as indicating the concerned area from a radiograph or giving a treatment plan based on predicted outcomes.

e. Continuous learning and adaptation:

-Model retraining: As new data available input (e.g., new patient cases, updated guidelines), ML models should be periodically retrained to maintain their accuracy, relevance and consistency with up-to-date clinical knowledge and practices.

-Feedback loops: Incorporating feedback from clinicians and patients to refine and improve model performance. Continuous feedback helps in identifying errors, updating models and enhancing the practicality of ML tools in clinical settings.

g. Clinical validation and approval:

-Clinical trials and validation: Before deploying ML models in real-world dental settings, rigorous clinical validation must undergo under condition involving clinical trials, pilot studies or comparative studies with existing diagnostic methods to ensure safety, efficacy and reliability. [19]

-Data security and compliance with regulations: As dentists and researchers harness clinical data for ML modeling, adherence to relevant data protection regulations is imperative. Ensuring compliance

with laws such as GDPR [20], HIPAA [21] or CCPA [22] is crucial to maintain confidentiality and keep sensitive information safe from unauthorized access.

h. Interpretability and explainability [23]:

-Model transparency: Interpretable models allow dentist to clearly understand how models reach the specific decisions especially in critical areas such as diagnosis and treatment planning.

-Explainable AI (XAI): aims to develop a suite of ML techniques so that can produce more explainable models while maintaining a high level of learning performance (e.g., prediction accuracy) [24], particularly for deep learning models often considered as "black boxes", using pathway visualization or feature highlighting in a diagnosis.

i. Patient-centric [25]care:

-Personalization: to create ML-personalized treatment plans according to patient's individual data such as genetic information, medical history, lifestyle and specific dental conditions.

-Improving patient outcomes: the ultimate goal is to provide faster and more efficient care that directly enhance patient outcomes [26].

References

1. Uribe SE, Sofi-Mahmudi A, Raittio E, Maldupa I, Vilne B. Dental Research Data Availability and Quality According to the FAIR Principles. J Dent Res 2022:101(11):1307-13.
2. Mohammad-Rahimi H, Rokhshad R, Bencharit S, Krois J, Schwendicke F. Deep learning: a primer for dentists and dental researchers. Journal of Dentistry 2023:130:104430.
3. Rad AE, Mohd Rahim MS, Rehman A, Altameem A, Saba T. Evaluation of current dental radiographs segmentation approaches in computer-aided applications. IETE Technical Review 2013:30(3):210-22.
4. Costa ED, Gaeta-Araujo H, Carneiro JA, Zancan BAG, Baranauskas JA, Macedo AA, Tirapelli C. Development of a dental digital data set for research in artificial intelligence: the importance of labeling performed by radiologists. Oral Surg Oral Med Oral Pathol Oral Radiol 2024:138(1):205-13.
5. Dima S, Wang KJ, Chen KH, Huang YK, Chang WJ, Lee SY, Teng NC. Decision Tree Approach to the Impact of Parents' Oral Health on Dental Caries Experience in Children: A Cross-Sectional Study. Int J Environ Res Public Health 2018:15(4).
6. Prakash M, Gowsika U, Sathiyapriya S. An Identification of Abnormalities in Dental with Support Vector Machine Using Image Processing. New Delhi: Springer India, 2015:29-40.
7. Prados-Privado M, Garcia Villalon J, Martinez-Martinez CH, Ivorra C, Prados-Frutos JC. Dental Caries Diagnosis and Detection Using Neural Networks: A Systematic Review. J Clin Med 2020:9(11).
8. Jain KR, Chauhan NC. Clustering Techniques for Dental Image Analysis. Dental Image Analysis for Disease Diagnosis. Cham: Springer International Publishing, 2019:103-28.
9. Leite AF, de Souza Figueiredo PT, Caracas H, Sindeaux R, Guimarães ATB, Lazarte L, de Paula AP, de Melo NS. Systematic review with hierarchical clustering analysis for the fractal dimension in assessment of skeletal bone mineral density using dental radiographs. Oral Radiology 2014:31(1):1-13.
10. Trella AL, Zhang KW, Nahum-Shani I, Shetty V, Doshi-Velez F, Murphy SA. Reward design for an online reinforcement learning algorithm supporting oral self-care. Proceedings of the AAAI Conference on Artificial Intelligence, 2023:15724-30.
11. Alvarez P. Deep Reinforcement Learning for Autonomous Dental Robotics. Periodontal Advancements: A Guide to the Latest in Gum Health 2021:70.
12. Hung M, Voss MW, Rosales MN, Li W, Su W, Xu J, Bounsanga J, Ruiz-Negron B, Lauren E, Licari FW. Application of machine learning for diagnostic prediction of root caries. Gerodontology 2019:36(4):395-404.
13. Sunaga M, Minabe M, Inagaki K, Kinoshita A. Effectiveness of a specially designed dental model for training, evaluation, and standardization of pocket probing. Journal of Dental Education 2016:80(12):1430-9.
14. Oliveira ALI, Baldisserotto C, Baldisserotto J. A Comparative Study on Machine Learning Techniques for Prediction of Success of Dental Implants. Berlin, Heidelberg: Springer Berlin Heidelberg, 2005:939-48.
15. Cejudo JE, Chaurasia A, Feldberg B, Krois J, Schwendicke F. Classification of Dental Radiographs Using Deep Learning. J Clin Med 2021:10(7).
16. Collins GS, Dhiman P, Ma J, Schlussel MM, Archer L, Van Calster B, Harrell FE, Jr., Martin GP, Moons KGM, van Smeden M, Sperrin M, Bullock GS, Riley RD. Evaluation of clinical prediction models (part 1): from development to external validation. BMJ 2024:384:e074819.
17. James G, Witten D, Hastie T, Tibshirani R. An introduction to statistical learning: Springer, 2013.

18. Ourang SA, Sohrabniya F, Mohammad-Rahimi H, Dianat O, Aminoshariae A, Nagendrababu V, Dummer PMH, Duncan HF, Nosrat A. Artificial intelligence in endodontics: Fundamental principles, workflow, and tasks. Int Endod J 2024.
19. Frantsve-Hawley J, Abt E, Carrasco-Labra A, Dawson T, Michaels M, Pahlke S, Rindal DB, Spallek H, Weyant RJ. Strategies for developing evidence-based clinical practice guidelines to foster implementation into dental practice. J Am Dent Assoc 2022:153(11):1041-52.
20. Goldsteen A, Ezov G, Shmelkin R, Moffie M, Farkash A. Data minimization for GDPR compliance in machine learning models. AI and Ethics 2022:2(3):477-91.
21. Riad AKI, Barek MA, Rahman MM, Akter MS, Islam T, Rahman MA, Mia MR, Shahriar H, Wu F, Ahamed SI. Enhancing HIPAA Compliance in AI-driven mHealth Devices Security and Privacy. 2024 IEEE 48th Annual Computers, Software, and Applications Conference (COMPSAC): IEEE, 2024:2430-5.
22. Mulgund P, Mulgund BP, Sharman R, Singh R. The implications of the California Consumer Privacy Act (CCPA) on healthcare organizations: Lessons learned from early compliance experiences. Health Policy and Technology 2021:10(3):100543.
23. Marcinkevičs R, Vogt JE. Interpretable and explainable machine learning: A methods-centric overview with concrete examples. WIREs Data Mining and Knowledge Discovery 2023:13(3).
24. Arrieta AB, Díaz-Rodríguez N, Del Ser J, Bennetot A, Tabik S, Barbado A, García S, Gil-López S, Molina D, Benjamins R. Explainable Artificial Intelligence (XAI): Concepts, taxonomies, opportunities and challenges toward responsible AI. Information fusion 2020:58:82-115.
25. Sharma NS. Patient centric approach for clinical trials: current trend and new opportunities. Perspectives in clinical research 2015:6(3):134-8.
26. Martin J, Mills S, Foley ME. Innovative models of dental care delivery and coverage: patient-centric dental benefits based on digital oral health risk assessment. Dental Clinics 2018:62(2):319-25.
